# Dual dye-loaded Au@Ag coupled to a lateral flow immunoassay for the accurate and sensitive detection of *Mycoplasma pneumoniae* infection†

**DOI:** 10.1039/c8ra03323d

**Published:** 2018-06-11

**Authors:** Xiaofei Jia, Chongwen Wang, Zhen Rong, Jian Li, Keli Wang, Zhiwei Qie, Rui Xiao, Shengqi Wang

**Affiliations:** College of Life Sciences & Bio-Engineering, Beijing University of Technology Beijing 100124 P. R. China sqwang@bmi.ac.cn +86-10-66932211; Beijing Institute of Radiation Medicine Beijing 100850 P. R. China ruixiao203@sina.com +86-10-66931422-5; Chinese PLA General Hospital Beijing 100853 P. R. China

## Abstract

We present an attractive model of surface-enhanced Raman scattering-based lateral flow immunoassay (SERS-LFIA) for the sensitive and accurate detection of *Mycoplasma pneumoniae* (MP) infection in human serum. The SERS-LFIA strip uses Au@Ag nanoparticles (Au@Ag NPs) loaded with two layers of Raman dye 5,5′-dithiobis-(2-nitrobenzoic acid) (DTNB) as SERS tags. The advantages of the dual dye-loaded SERS tags (Au/DTNB@Ag/DTNB) are the high sensitivity and the bioconjugation flexibility of the detection antibody. As determined from our SERS-LFIA strip, human IgM was quantified by monitoring the SERS signal on the test line. The limit of detection for human IgM was 0.1 ng mL^−1^, which was 100 times more sensitive than that by using the colorimetric method. Our assay results for 20 MP-specific IgM positive serum specimens showed 100% accuracy and detection rate, whereas the parallel enzyme-linked immunosorbent assay only showed 85% detection rate. The SERS-LFIA strip also exhibited high specificity and potential clinical applications. Therefore, our SERS-based LFIA strip has strong potential for practical applications in the sensitive and rapid detection of MP.

## Abstract 2

Schematic illustration of quantitative detection of human IgM using SERS-based lateral flow immunoassay.

## Graphical abstract

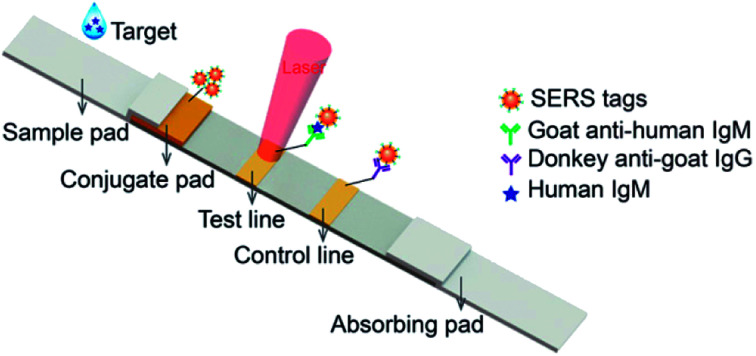

## 1. Introduction

*Mycoplasma pneumoniae* (*M. pneumoniae*, MP), a unique pathogen lacking a peptidoglycan cell wall and self-reproducing without a host, is the common reason of pneumonia and respiratory diseases in all age groups worldwide.^1–3^ Approximately 6–30% of pneumonia cases in all ages can be ascribed to *M. pneumoniae* infection, and the epidemic peaks in intervals of 3–7 years.^4,5^ The early diagnosis of *M. pneumoniae* infection is important for deciding the treatment modality and guiding the appropriate antibiotic therapy.^6^ The current gold standard method remains conventional culture, which is the most definitive and inexpensive diagnosis method for *M. pneumoniae*.^3^ However, MP culture is time consuming (2–8 weeks) and requires professional media and trained personnel.^7^ Serological analysis, another conventional method, is uncertain for the definitive identification of *M. pneumoniae* infection on account of its low diagnostic sensitivity.^3,8^ As an alternative, numerous molecular methods based on antigen detection or nucleic acid have appeared as major techniques for the identification of *M. pneumoniae*; these methods contain DNA sequencing, polymerase chain reaction (PCR), and enzyme-linked immunosorbent assays (ELISA).^4,9–11^ These molecular techniques have achieved specificity and sensitivity in the detection of *M. pneumoniae* infection but also exhibited some shortcomings, including the requirement of sophisticated equipment or expensive reagent, tedious or complex procedures, and the occasional false-positive results.^3^

A membrane-based lateral flow immunoassay (LFIA) strip has been extensively applied in many fields due to its simplicity, flexibility, rapidity, and use of low-cost strips; in this method, nanoparticles (NPs) are combined with chromatography and immunochemical reactions.^12,13^ The principle of most LFIA tests is based on color visualization using small size Au NPs as reporters.^14,15^ Nevertheless, these common LFIA strips have poor sensitivity and can only provide qualitative and semi-quantitative result on analyte concentrations, thus limiting their application.^16,17^ Novel signal-enhancement strategies, including dual Au NPs-based signal enhancement strategy, enzyme-amplified signal system, fluorescence-based signal enhancement method, and magnetic NP-based strategy have been developed to overcome these disadvantages.^18–21^ However, in these strategies, the results may undergo optical interference, and additional steps are required for signal detection.

A surface-enhanced Raman scattering (SERS)-based LFIA technology has recently attracted more and more attention because of its high sensitivity and quantitative analysis potential.^22–26^ The fundamental principle of this technique is using functional SERS tags instead of the Au NPs, which are utilized in conventional LFIA strips, such as the Raman reporter-labeled and antibody-conjugated metal NPs.^27–29^ The incident light field can be significantly enhanced by the localized surface plasmon effects when the Raman reporter molecules coupled on the SERS tags exposure to a single excitation light source.^30–33^ This enhancement impact displays promise in resolving the poor sensitivity problem of conventional LFIA and luminescence or fluorescence-based assay techniques.^22^ Furthermore, quantitative analysis can be performed in Raman spectroscopy because the intensity of the SERS signal is directly in accordance with the number of SERS tags on the test line.^19,34,35^ The combination of SERS tags and LFIA contributes to the sensitivity and accuracy for quantitative point-of-care test (POCT). This new combined biosensor system is helpful to practical use in clinical diagnostics.

In our research, we reported a new SERS-LFIA strip for the quantitative and highly sensitive detection of human IgM and the accurate early diagnosis of MP infection in human serum. MP-specific IgM antibodies appear early at the onset of the infection, peak in a few of weeks, then drop to extremely low levels. Because of the early occurrence and short life span of IgM antibodies, the detection for MP-specific IgM allows the diagnosis of early and acute infection using a single serum specimen.^20^ Approximately 44 nm Au@Ag core–shell NPs were utilized as the SERS substrates because Au@Ag has better SERS activity than same-sized Au NPs and a more uniform distribution than Ag colloids.^36,37^ These characteristics are critical to signal strength and reproducibility. The Au@Ag NPs were loaded with two layers of DTNB molecules as the dual-dye SERS tags. Owing to the strong enhancement capability of plasmonic Au@Ag NPs, the dual dye-loaded SERS tags exhibited strong and quantitative SERS signal and were subsequently conjugated with target detection antibody to serve as the SERS tags in the LFIA strip.^38^ Under the optimized condition, the SERS-LFIA strip became a rapid and effective detection tool for the detection of human IgM and the limit of detection (LOD) was as low as 0.1 ng mL^−1^. This LOD value is 100 times more sensitive than that acquired from the colorimetric method. Moreover, we compared the assay results for 20 MP clinical positive specimens with those obtained from a commercially available semi-quantitative MP-specific IgM ELISA kit to validate our proposed strip for clinical application. So far as we know, this research is the first to utilize SERS-based LFIA strip to accomplish MP rapid and highly sensitive detection. This approach can be a suitable diagnostic tool for MP detection.

## 2. Experimental section

### 2.1. Chemicals and materials

Chloroauric acid (HAuCl_4_), ascorbic acid (AA), silver nitrate (AgNO_3_), 5,5′-dithiobis-(2-nitrobenzoic acid) (DTNB), *N*-(3-dimethylaminopropyl)-*N*′-ethylcarbodiimide hydrochloride (EDC), *N*-hydroxysulfosuccinimide sodium salt (sulfo-NHS), polyvinylpyrrolidone (PVP, 40 K), Tween-20, bovine serum albumin (BSA), goat anti-human IgM, goat anti-mouse IgG, and IgM from human serum (human IgM) were obtained from Sigma-Aldrich (USA). Mouse anti-human IgM (μ-chain specific) was purchased from Fapon Biotech Inc., (China). Donkey anti-goat IgG was obtained from Sangon Biotech Co., Ltd., (Shanghai). MP P1 antigen was purchased from Xiamen One Clone Biotech Inc., (China). Sodium chloride (NaCl), sucrose, sodium tetraborate (Na_2_B_4_O_7_·10H_2_O), and boric acid were acquired from Sinopharm Chemical Reagent Co., Ltd., (China). 1 M Tris–HCl (pH 8.0) was obtained from Applygen Technologies Inc., (China). The nitrocellulose (NC) membrane (Hi-flow plus HF180) with 6 μm pore size was obtained from Millipore Corporation (USA). The glass fiber sample loading pad (XQ-Y2), glass fiber conjugate pad (GL0194), PVC bottom plate and absorbent pad were obtained from Jieyi Biotechnology Co., Ltd., (China).

Clinical serum specimens were collected from Chinese PLA General Hospital. Informed consent was written by all patients. All procedures were performed according to the approved guidelines of the Ethics Committee of the Institute of Radiation Medicine and Chinese PLA General Hospital. Each subject was phlebotomized to collect 3 mL of whole blood. Serum was acquired by centrifugation. Serum specimens were stored at −80 °C.^39^

### 2.2. Instruments

High-magnification transmission electron microscopy (TEM) images were acquired with a Hitachi H-9000 TEM with an acceleration voltage of 100 kV. UV-Vis spectra were obtained by a Shimadzu 2600 spectrometer. Dynamic light scattering (DLS) data for the NPs were obtained by a Nano-ZS90 (Malvern) apparatus. Raman spectra and SERS mapping images of the tested SERS-LFIA strips were recorded on a Renishaw inVia plus Raman system with an excitation laser at 785 nm. Incident radiation coupled to an Olympus BX51 optical microscope was focused to a 2 μm diameter spot *via* a 5× objective. The acquisition time of the data at each spot was 5 s. The signal intensities were standardized in regard to the silicon wafer at 520 cm^−1^.^40^

### 2.3. Synthesis of Au/DTNB@Ag/DTNB NPs

Au NPs were prepared according to the citrate reduction method. In brief, 100 mL of HAuCl_4_ solution (0.01%, w/v) was first heated to the boiling point with stirring. Afterward, 1.5 mL of trisodium citrate (1%, w/v) was added rapidly to the boiling solution. The suspension was boiled for 15 min and then allowed to reach thermal equilibrium at room temperature, which yielded the Au NPs with a diameter of 35 nm.

DTNB-modified Au NPs (Au/DTNB NPs) were prepared by attaching DTNB molecules on the Au NP surface. In brief, 10 μL of 10 mM DTNB ethanol solution was mixed to 10 mL of Au NPs solution. The mixture was under vigorous stirring at room temperature for 4 h. The ultimate solution was centrifuged at 8000 rpm for 8 min to eliminate the redundant DTNB molecules. The sediment was resuspended in 10 mL of deionized water.^41^

The Au/DTNB@Ag core–shell NPs were prepared by the following procedure. First, 10 mL of Au/DTNB NPs solution was four times diluted and then heated to boiling temperature. Subsequently, 0.5 mL of trisodium citrate (1%, w/v) was rapidly added into the solution under vigorous stirring. Ultimately, 1 mL of silver nitrate (1 mM) was drop-wise added into the above solution, and the resulting mixture was kept on boiling for another 15 min. Finally, the color of solution changed from red to orange red, which indicated the formation of Au/DTNB@Ag NPs.

The preparing method of DTNB-modified Au/DTNB@Ag NPs (Au/DTNB@Ag/DTNB) is similar to that of the DTNB-modified Au NPs.

### 2.4. Preparation of Au/DTNB@Ag/DTNB NP SERS tags for SERS-based lateral flow immunoassay

The dual dye-loaded SERS tags (Au/DTNB@Ag/DTNB-antibody) were prepared through the EDC/sulfo-NHS coupling between the amino group of the antibody and DTNB–COOH group. The SERS tags for detecting human IgM were prepared by conjugating goat anti-human IgM, and the SERS tags for the specific recognition of MP-specific IgM were prepared by conjugating mouse anti-human IgM (μ-chain specific). In brief, 500 μL of Au/DTNB@Ag/DTNB NPs were reacted with 20 μL of EDC/sulfo-NHS solution (1 mg mL^−1^), and the reaction solution was under vigorous shaking for 15 min. Afterward, 5 μL of the antibody (5 mg mL^−1^) and 500 μL of the BBS buffer (borate buffer solution, 10 mM, pH 8.0) were mixed into the above solution, and the resulting solution was under vigorous shaking for 2 h for conjugation.^41^ The unreacted carboxyl sites of SERS tags were blocked with 100 μL of BSA (10%, w/v) for an additional 1 h. The precipitate was separated by centrifugation and then washed several times with BBS buffer (10 mM, pH 8.0). The resultant precipitate was resuspended with 500 μL of preservation solution containing Tris–HCl (50 mM, pH 8.0), 1% BSA (w/v), 0.1% PVP (w/v), 10% sucrose (w/v), and 0.5% Tween-20 (v/v) for further use.

### 2.5. Preparation of SERS-LFIA strips

**Fig. 1:**
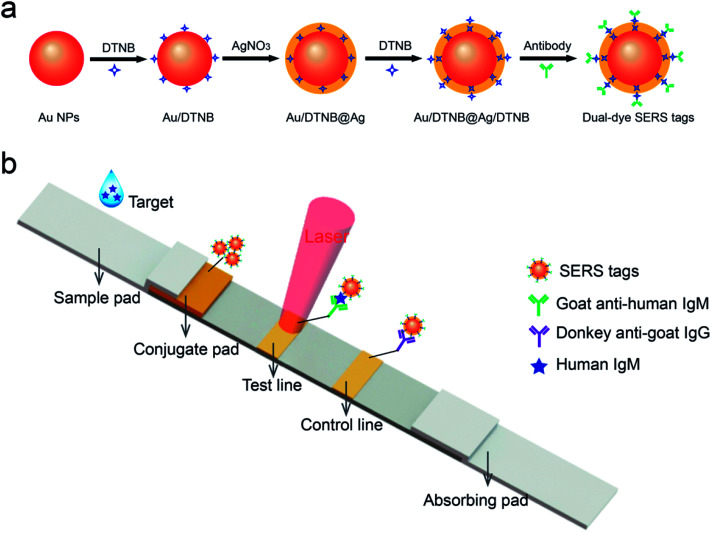
(a) Synthetic route for dual dye-loaded SERS tags; (b) schematic illustration of quantitative detection of human IgM using SERS-based lateral flow immunoassay.

The SERS-based LFIA strip comprised an absorbent pad, an NC membrane with 6 μm pore size, a conjugate pad with SERS tags and a sample loading pad (Fig. 1b). To ensure solution migration, four components was affixed to a plastic backing card in sequence. A desired volume of the as-prepared SERS tags was dropped on the conjugate pads. The conjugate pads with SERS tags were dried at 37 °C incubator for 3 h. For detecting human IgM, the test and control zones of the NC membrane were coated by spraying 1 mg mL^−1^ of goat anti-human IgM and 1 mg mL^−1^ of donkey anti-goat IgG, separately. For the specific recognition of MP-specific IgM, the test and control lines were prepared by spraying MP P1 antigen and goat anti-mouse IgG, respectively. The NC membrane was prepared by spraying antibody or antigen at a rate of 1 μL cm^−1^ using a spraying platform (Biodot xyz5050). In the end, the integrated SERS-LFIA strips were stored in an airtight container.

## 3. Results and discussion

### 3.1. Principle of SERS-based LFIA

Fig. 1b shows the testing principle for quantitative analysis of human IgM antibody based on the SERS-LFIA strip. This experiment is based on the formation of antibody/antigen/antibody-conjugated SERS tags sandwich immune complexes. The sample solution containing target human IgM was dropped on the sample pad. Specific recognition and combination occurred between the human IgM- and anti-human IgM-conjugated SERS tags when the sample solution travelled through the conjugation pad. Under the capillary action, the resulting immunocomplexes continued migrating along the NC membrane and were caught by the anti-human IgM antibodies, which were previously coated on the test line. The excess SERS tags continued migrating and were caught by donkey anti-goat IgG immobilized on the control zone of NC membrane. The aggregation of SERS tags on the test/control line produced two visible red bands. When there was no target human IgM existing in the sample solution, only the red band of the control zone was observed. The line also validated the test system well. Finally, the SERS signal of the test line was tested for the quantitative analysis of human IgM.

### 3.2. Characterization of Au/DTNB@Ag/DTNB SERS tags

SERS tags are usually applied in label method as the extrinsic mode of target detection and have three basic components: a metal nanostructure as enhancing substrate, a specific Raman reporter molecule to produce specific SERS signal, and biorecognition molecules to identify target substance. In this work, a dual dye-loaded Au/DTNB@Ag/DTNB nanostructure was designed as a novel SERS tag that possessed two advantages over the commonly used Au or Ag NP SERS tags [Fig. 1a]. Au/DTNB@Ag/DTNB consists of two layers of DTNB molecules. DTNB acted as the Raman reporter molecule that was modified on the surface and embedded in the Au@Ag NPs to generate stable and strong SERS signal. The outside DTNB layer of Au/DTNB@Ag/DTNB NPs was biofunctional to serve as a Raman reporter and link with amino groups of the antibodies.

**Fig. 2:**
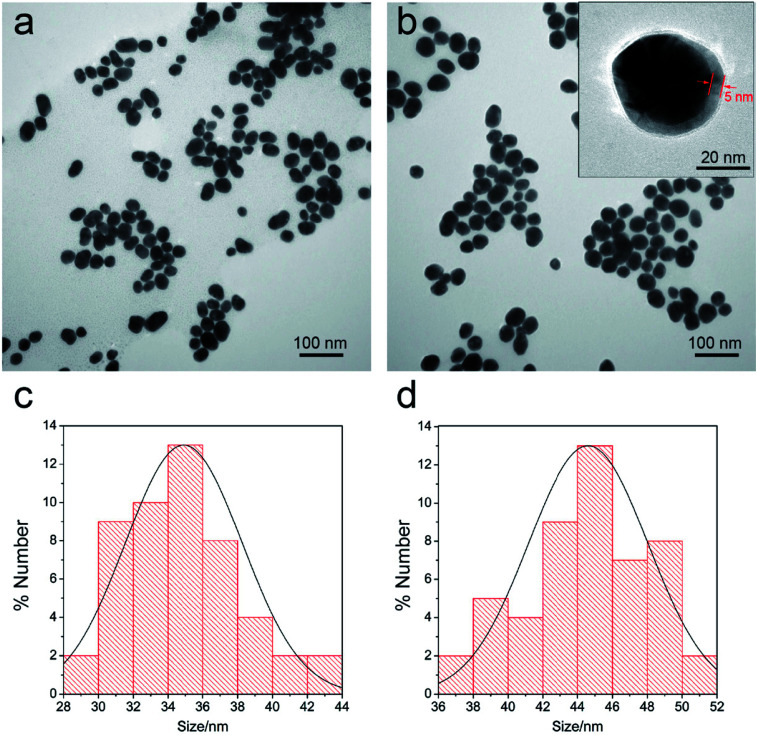
TEM images of the prepared Au NPs (a) and the Au/DTNB@Ag NPs (b). HRTEM image of the magnified Au/DTNB@Ag NPs was inset in (b) (arrows indicate the thickness of Ag shell). DLS distributions of the prepared Au NPs (c) and Au/DTNB@Ag NPs (d).

The Au@Ag core–shell NPs were prepared by *in situ* growth of Ag shells on the surface of Au NPs. In this study, the average diameter of the synthesized Au NPs was 35 nm. The Au NPs were utilized as the seeds and the Raman dye DTNB could be easily absorbed onto the Au surface *via* the disulfide bond in the DTNB molecules. In brief, 1 mL of AgNO_3_ solution (10 mM) was used to produce an Ag shell over the 100 mL of Au seed solution. The synthesized Au seeds and Au@Ag NPs were characterized *via* TEM (Fig. 2a and b). The high-resolution (HR) TEM image of Au@Ag NPs denotes the thickness of the Ag shell was approximately 5 nm as inset in Fig. 2b. The DLS data in Fig. 2c and d also show that the average diameter of Au NPs increased from 35 nm to approximately 44 nm after Ag shell coating.

### 3.3. Dual dye-loaded Au@Ag NPs

**Fig. 3:**
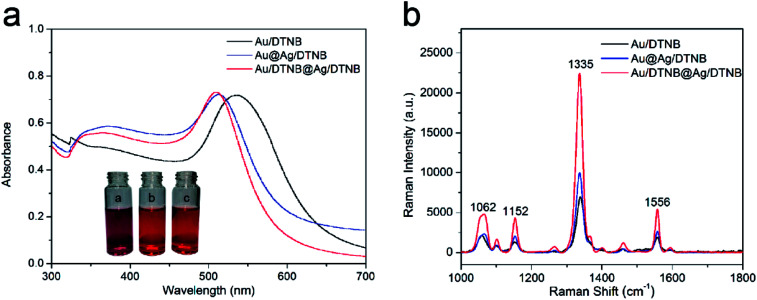
(a) UV-visible spectra of Au/DTNB, Au@Ag/DTNB, and Au/DTNB@Ag/DTNB NPs. The insets show the photographs of the three kinds of SERS tags. (b) SERS intensity of Au/DTNB, Au@Ag/DTNB, and Au/DTNB@Ag/DTNB NPs under the same conditions.

Other two kinds of single-dye modified SERS tags (Au/DTNB and Au@Ag/DTNB NPs) were synthesized for comparison to verify the SERS activity of the newly designed dual dye-loaded SERS tags. These SERS tags were modified with the same concentration Raman dye molecule DTNB under magnetic stirring for 4 h. Fig. 3a reveals the UV-Vis spectra of the prepared SERS tags. The extinction peak of the Au NPs appeared at the wavelength of 525 nm. This peak blue shifted at approximately 23 nm after the Ag shell coating. The UV-Vis spectra of Au/DTNB@Ag/DTNB appeared almost identical with those of Au@Ag/DTNB NPs, indicating that embedding DTNB inside the Ag shell did not influence the wavelength of Au@Ag NPs. The Raman spectra of the three kinds of SERS tags as shown in Fig. 3b. The main Raman band of DTNB centered at 1335 cm^−1^ was used for comparison. The SERS signal of our proposed dual dye-loaded SERS tags (Au/DTNB@Ag/DTNB) was twice those of Au/DTNB NPs and about 1.5 times those of Au@Ag/DTNB NPs.

### 3.4. Quantitative analysis of human IgM based on SERS-LFIA assay

**Fig. 4:**
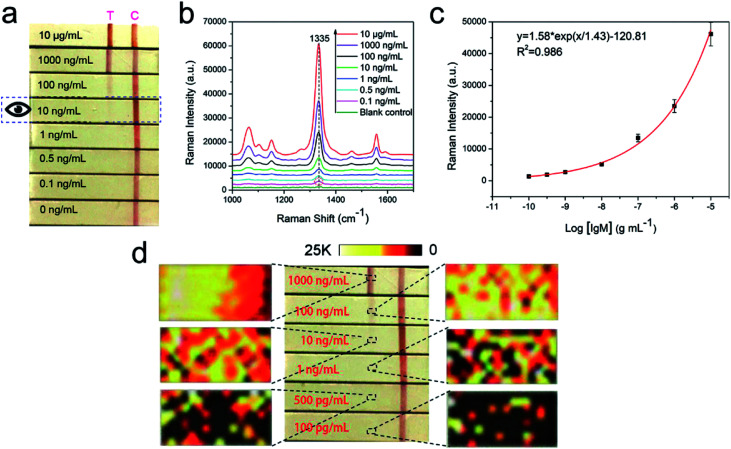
(a) Photographs of SERS-LFIA strips based on Au/DTNB@Ag/DTNB tags after applying different concentrations of human IgM. (b) SERS spectra measured in the corresponding test lines. The excitation laser energy was 10 mW, and the integration times were 5 s. (c) Calibration curve for human IgM at a concentration of 0.1 ng mL^−1^ to 10 μg mL^−1^ obtained by using SERS intensity at 1335 cm^−1^. Error bars represent the standard deviations from five independent measurements. (d) SERS mapping images acquired using SERS signal intensity at Raman peak 1335 cm^−1^ for six different human IgM concentrations in 0.1–1000 ng mL^−1^.

To confirm the detection sensitivity of the proposed SERS-LFIA strips, we used goat anti-human IgM-conjugated Au/DTNB@Ag/DTNB NPs as models for capture and reporting agents. The SERS detection protocol is shown in Fig. 1b. The human IgM was diluted in a range of 10 μg mL^−1^ to 0.1 ng mL^−1^, and 1× PBST (1× PBS, 0.05% Tween-20, pH 7.4) as a blank control. Fig. 4a shows the photographs of the SERS-LFIA strips testing for human IgM in different concentrations (0.1 ng mL^−1^ to 10 μg mL^−1^). When the human IgM concentration increased, many immunocomplexes were formed, and the visible red color was found on the test line. The control line of each strip displayed a red band, indicating that the SERS-LFIA strip worked properly. The color observed on the test line was at a higher concentration of human IgM (10 ng mL^−1^). Such high visual detection result could not meet the demand for actual testing and provide quantitative information on target concentrations. However, for the SERS-based LFIA strip, it is feasible that human IgM is quantitatively analyzed through the characteristic Raman signal of the Raman dye molecules of SERS tags on the test line. Fig. 4b shows the SERS spectra of different concentrations of target human IgM. The SERS signals on the test lines were weakened when the human IgM concentration decreased, and the main peak at 1335 cm^−1^ was distinguished when the human IgM concentration declined to 0.1 ng mL^−1^. Therefore, the LOD of the proposed SERS-LFIA strips based on the dual dye-loaded SERS tags was 0.1 ng mL^−1^. The calibration curve was plotted between the logarithm of human IgM concentrations (0.1 ng mL^−1^ to 10 μg mL^−1^) and the SERS signal intensity at Raman peak 1335 cm^−1^, and is shown in Fig. 4c. A good linear relationship was observed in the range of 0.1 ng mL^−1^ to 10 μg mL^−1^ human IgM concentration (*R*^2^ = 0.986). The homogeneity of SERS signals across the test line was observed by mapping the SERS spectra over an extended area (800 μm × 400 μm) with a 50 μm step at Raman peak 1335 cm^−1^. For different concentrations (0.1–1000 ng mL^−1^) of human IgM, the corresponding SERS mapping images across the test lines were obtained as shown in Fig. 4d. The SERS signal intensity at the front of the test area is higher due to the fact that it is the first place the fluid encounters as it migrates through the strip at high human IgM concentration (1000 ng mL^−1^). The homogeneity of SERS signals across the test line decreases with the IgM concentration decreasing from 100 ng mL^−1^ to 100 pg mL^−1^.

The repeatability of SERS signals was measured at 10 different spots on the three different batches of SERS-LFIA strips, and the result is shown in Fig. S1.† The relative standard deviation from was 10.6%, which indicated the good reproducibility of the SERS-LFIA strips. We further compared the sensitivity of SERS-LFIA strip based on the dual dye-loaded SERS tags with that of other two SERS-LFIA strips based on single dye-loaded SERS tags (Au/DTNB-antibody or Au@Ag/DTNB-antibody). Fig. S2† shows that the visual and SERS signal detection limits of Au/DTNB- and Au@Ag/DTNB-based LFIA strips were 10 and 1 ng mL^−1^, severally. Thus, the detection sensitivity of the Au/DTNB@Ag/DTNB based on SERS-LFIA strip was dramatically enhanced by nearly 10 and 100 folds in comparison with those of single-dye-loaded SERS tag-based and conventional LFIA, respectively.

### 3.5. Detection rate of MP-specific IgM positive serum specimens based on SERS-LFIA strips

**Fig. 5:**
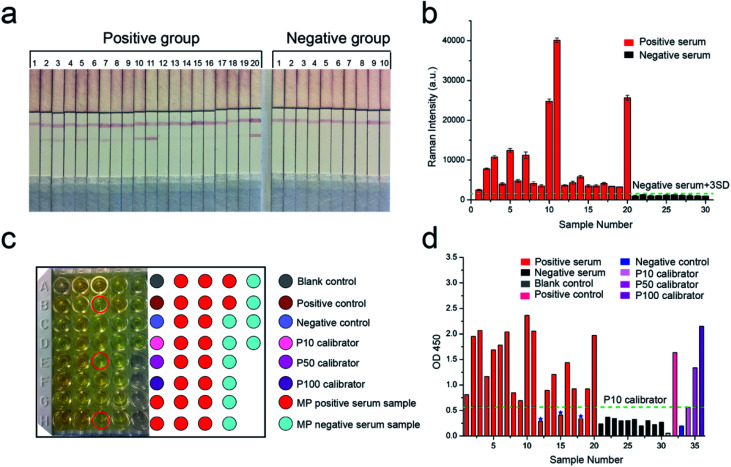
(a) Photographs of SERS-LFIA strips applied in serum specimens. The numbers above the strips represent 20 MP-specific IgM positive serum specimens and 10 MP-specific IgM negative serum specimens. (b) SERS signal intensities on the test lines of the corresponding SERS-LFIA strips. The error bars show standard deviation from five independent measurements. (c) ELISA for 20 MP-specific IgM positive serum specimens, 10 MP-specific IgM negative serum specimens, serum diluent (blank), positive control, negative control, and three calibrators (P10, P50, and P100) in a 96-well plate. The red circles represent three MP-specific IgM positive serum specimens that did not lead to the change of the color to darker yellow. (d) Absorbance of these specimens at 450 nm.

MP-specific IgM positive clinical serum specimens were detected to confirm the effectiveness and sensitivity of our proposed SERS-LFIA strips for MP clinical serum specimens. According to the methods described earlier, the test and control lines were prepared by spraying MP P1 antigen and goat anti-mouse IgG, respectively, for the specific recognition of MP-specific IgM. Twenty MP-specific IgM positive clinical serum specimens and ten MP-specific IgM negative clinical serum specimens were confirmed by using the diagnostic kit for measurement of antibodies to *Mycoplasma pneumoniae* (passive particle agglutination), SERODIA-MYCO II from Fujirebio Inc. (Japan). In brief, 10 μL of clinical serum and 60 μL of 2% NaCl (w/v, pH 7.0) solution were mixed and dropped on the sample pad of the prepared SERS-LFIA strips. Based on immunoreaction and chromatography, the MP-specific IgM positive serum specimens would develop both red T and C lines visible to the naked eye, but MP-specific IgM negative serum specimens would only develop visible red C lines. As shown in Fig. 5a, the test lines of the number 2, 3, 5, 7, 10, 11, 14, and 20 of the positive group were readily visible, whereas the others were not easily observed by the naked eye. All the test lines of the negative group were not observed.

The SERS signal intensities at the Raman peak of 1335 cm^−1^ on the test lines of the SERS-LFIA strips were measured and analyzed and is shown in Fig. 5b. The SERS signal intensities of all the MP-specific IgM positive serum samples were stronger than those of the MP-specific IgM negative serum samples. The SERS signal intensities of 10 MP-specific IgM negative serum samples were low, which indicated that the proposed SERS-LFIA strips have high specificity for the MP-specific IgM positive serum. The result of statistical analysis shows that the Raman intensity of positive group is observably higher than the negative group (*p < 0.05) as shown in Fig. S3.† The cut-off value (COV, negative serum + 3SD) was calculated as the averaged signal intensities of 10 MP-specific IgM negative serum specimens to three times of standard deviation measurements, and the obtained value was 1543. The serum specimens with the SERS signal intensities ≥1543 were considered MP positive, whereas those with intensities <1543 were considered MP negative. The SERS signal intensities of MP-specific IgM positive serum specimens were stronger than those of MP-specific IgM negative serum specimens and higher than the COV. With the proposed SERS-based LFIA strips, the detection rate of MP-specific IgM positive serum specimens was 100%.

ELISA was conducted to compare the sensitivity of our proposed SERS-LFIA strips with the common detection sensitivity provided by a standardized immunoassay format. The same MP-specific IgM positive serum specimens and negative serum specimens were performed using SeroMP™ IgM kit (Savyon® Diagnostics Ltd, Israel). A 96-well plate was coated with MP P1 antigens that capture the MP-specific IgM antibodies in MP-specific IgM positive serum. MP-specific IgM antibodies were bound to the capture antigens, whereas HRP-conjugated anti-human IgM (detecting antibodies) was bound to MP-specific IgM antibodies. When the enzyme-linked immunocomplexes were formed, a TMB-substrate was added, and the reaction was terminated using stop solution (1 M H_2_SO_4_). The last step resulted in the color changing from yellow to dark yellow as shown in Fig. 5c. The right color chart represents the position of sample wells. The color caused by most MP-specific IgM positive serum specimens (except for three serum specimens marked with red circle) was darker than those of 10 MP-specific IgM negative serum specimens. The absorbance at 450 nm converted by the enzyme was measured in a microplate reader (Bio Tek Instruments, Inc., USA) and analyzed (Fig. 5d). The absorbance of blank control was the lowest, which was consistent with the lightest yellow color in Fig. 5c. The absorbance of negative control was mildly higher than the blank control. The absorbance values of positive control, P100 calibrator (representing high MP positive human serum), P50 calibrator (representing medium MP positive human serum), and P10 calibrator (representing low MP positive human serum) were also reasonable. According to the instructions, the cut-off value was the absorbance of P10 calibrator at 450 nm and the obtained value was 0.56. The serum specimens with the absorbance values higher than that of the P10 calibrator were considered MP positive and lower than P10 were considered MP negative. The absorbance of 10 MP negative serum specimens was lower than that of the P10 calibrator. The absorbance of 20 MP positive serum specimens should be higher than the P10 calibrator. However, the absorbance values of three serum samples 12, 15, and 18 were lower than that of the P10 calibrator. The detection rate of MP-specific IgM positive serum specimens based on ELISA was 85%. The above results indicated the sensitivity of the SERS-based LFIA was better than that of ELISA.

### 3.6. Specificity of SERS-LFIA strip for MP-specific IgM positive serum

**Fig. 6:**
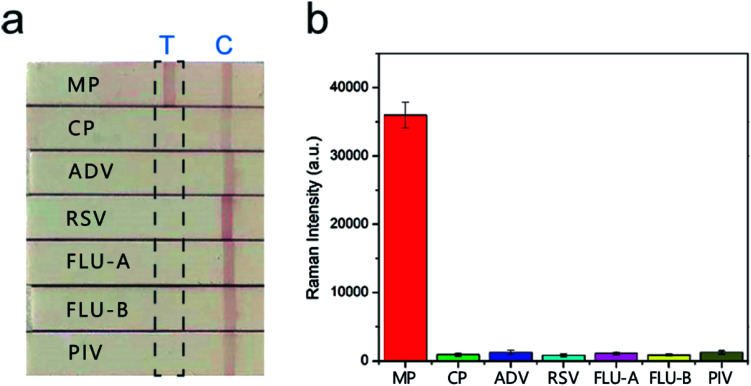
Specificity of the proposed SERS-LFIA strips. (a) The SERS-LFIA strip was used to test MP-specific IgM positive serum as testified by (b) strong Raman signal intensity on the test line, whereas negative results were obtained from other sera. Error bars are the standard deviation of five independent experiments.

The sera infected with the other six respiratory tract pathogens, *Chlamydia pneumoniae* (CP), adenovirus (ADV), respiratory syncytial virus (RSV), influenza A (FLU-A), influenza B (FLU-B), and parainfluenza (PIV) were diagnosed by commercial serology kits. These sera were also tested with the SERS-based LFIA strips to further evaluate their specificity and selectivity. The visual detection results of the strips in Fig. 6a show that two red bands were observed for MP positive serum, whereas only the control line was observed for the other six sera. The SERS detection results (Fig. 6b) also indicated that these sera only introduced weak Raman signals on the test lines, whereas MP positive serum exhibited a strong signal. Moreover, the SERS signal intensities of the other six sera were lower than the COV. These sera were confirmed negative, and no obvious cross-reaction was detected. The results proved that our SERS-LFIA strip possessed good specificity and selectivity for detecting the MP-specific IgM positive serum.

We compared the characteristics of our proposed SERS-based LFIA strip using the dual dye-loaded SERS tags with other recently reported LFIA strip for respiratory pathogens detection. Table S1† shows that our results are superior or equivalent to the results of other methods.^19,42–45^

## 4. Conclusions

In this study, a rapid, sensitive, and specific SERS-based LFIA strip was developed for the early diagnosis of MP detection. Instead of the Au colloids employed in a typical LFIA strip, dual DTNB-labeled Au@Ag NPs were utilized as SERS tags in the SERS-LFIA biosensor to allow detection and quantification through SERS signal intensity from the test line. Based on this strategy, human IgM was quantitatively and sensitively detected, and MP infection was accurately diagnosed in clinical samples. The LOD of the proposed SERS-LFIA strip for human IgM was estimated to be 0.1 ng mL^−1^, which are 100 folds more sensitive than that by using the aggregation-based colorimetric method. The detection rate of 20 MP positive serum specimens based on our SERS-LFIA strip was up to 100% and 15% higher than that of parallel ELISA. In addition, this strip displayed many other merits, including short assay time, simple operating procedure, and high specificity and reproducibility. We believe that SERS-LFIA platform has a good potential in field applications for the rapid diagnosis of many diseases.

## Conflicts of interest

There are no conflicts to declare.

## Supplementary Material

RA-008-C8RA03323D-s001

## References

[cit1] Balish M. F., Distelhorst S. L. (2016). Front. Microbiol..

[cit2] Meyer Sauteur P. M., Unger W. W., Nadal D., Berger C., Vink C., van Rossum A. M. (2016). Front. Microbiol..

[cit3] Diaz M. H., Winchell J. M. (2016). Front. Microbiol..

[cit4] Qu J., Gu L., Wu J., Dong J., Pu Z., Gao Y., Hu M., Zhang Y., Gao F., Cao B., C. Wang (2013). BMC Infect. Dis..

[cit5] Pereyre S., Goret J., Bebear C. (2016). Front. Microbiol..

[cit6] Lee S. C., Youn Y. S., Rhim J. W., Kang J. H., Lee K. Y. (2016). Medicine.

[cit7] Lai C. H., Chang L. L., Lin J. N., Chen W. F., Kuo L. L., Lin H. H., Chen Y. H. (2013). PLoS One.

[cit8] Stambach N. R., Carr S. A., Cox C. R., Voorhees K. J. (2015). Viruses.

[cit9] Dumke R., Jacobs E. (2016). Front. Microbiol..

[cit10] Zhao F., Liu L., Tao X., He L., Meng F., Zhang J. (2015). PLoS One.

[cit11] Yamazaki T., Kenri T. (2016). Front. Microbiol..

[cit12] Huang X., Aguilar Z. P., Xu H., Lai W., Xiong Y. (2016). Biosens. Bioelectron..

[cit13] Wang X., Choi N., Cheng Z., Ko J., Chen L., Choo J. (2017). Anal. Chem..

[cit14] Lee S., Mehta S., Erickson D. (2016). Anal. Chem..

[cit15] Zhang L., Huang Y., Wang J., Rong Y., Lai W., Zhang J., Chen T. (2015). Langmuir.

[cit16] Liu F., Zhang H., Wu Z., Dong H., Zhou L., Yang D., Ge Y., Jia C., Liu H., Jin Q., Zhao J., Zhang Q., Mao H. (2016). Talanta.

[cit17] Smits H. L., Abdoel T. H., Solera J., Clavijo E., Diaz R. (2003). Clin. Vaccine Immunol..

[cit18] Zhu J., Zou N., Zhu D., Wang J., Jin Q., Zhao J., Mao H. (2011). Clin. Chem..

[cit19] Maneeprakorn W., Bamrungsap S., Apiwat C., Wiriyachaiporn N. (2016). RSC Adv..

[cit20] Ou L., Lv Q., Wu C., Hao H., Zheng Y., Jiang Y. (2016). J. Microbiol. Methods.

[cit21] Duan D., Fan K., Zhang D., Tan S., Liang M., Liu Y., Zhang J., Zhang P., Liu W., Qiu X., Kobinger G. P., Fu Gao G., Yan X. (2015). Biosens. Bioelectron..

[cit22] Blanco-Covian L., Montes-Garcia V., Girard A., Fernandez-Abedul M. T., Perez-Juste J., Pastoriza-Santos I., Faulds K., Graham D., Blanco-Lopez M. C. (2017). Nanoscale.

[cit23] Cheng Z., Choi N., Wang R., Lee S., Moon K. C., Yoon S. Y., Chen L., Choo J. (2017). ACS Nano.

[cit24] Park M., Jung H., Jeong Y., Jeong K. H. (2017). ACS Nano.

[cit25] Sánchez-Purrà M., Roig-Solvas B., Versiani A., Rodriguez-Quijada C., de Puig H., Bosch I., Gehrke L., Hamad-Schifferli K. (2017). Mol. Syst. Des. Eng..

[cit26] Xie Y., Chang H., Zhao K., Li J., Yang H., Mei L., Xu S., Deng A. (2015). Anal. Methods.

[cit27] Hwang J., Lee S., Choo J. (2016). Nanoscale.

[cit28] Hu C., Shen J., Yan J., Zhong J., Qin W., Liu R., Aldalbahi A., Zuo X., Song S., Fan C., He D. (2016). Nanoscale.

[cit29] Shen J., Su J., Yan J., Zhao B., Wang D., Wang S., Li K., Liu M., He Y., Mathur S., Fan C., Song S. (2015). Nano Res..

[cit30] Li J. F., Huang Y. F., Ding Y., Yang Z. L., Li S. B., Zhou X. S., Fan F. R., Zhang W., Zhou Z. Y., Wu D. Y., Ren B., Wang Z. L., Tian Z. Q. (2010). Nature.

[cit31] Fu X., Cheng Z., Yu J., Choo P., Chen L., Choo J. (2016). Biosens. Bioelectron..

[cit32] Wang C., Wang J., Li P., Rong Z., Jia X., Ma Q., Xiao R., Wang S. (2016). Nanoscale.

[cit33] Su J., Wang D., Nörbel L., Shen J., Zhao Z., Dou Y., Peng T., Shi J., Mathur S., Fan C., Song S. (2017). Anal. Chem..

[cit34] Gao X., Zheng P., Kasani S., Wu S., Yang F., Lewis S., Nayeem S., Engler-Chiurazzi E. B., Wigginton J. G., Simpkins J. W., Wu N. (2017). Anal. Chem..

[cit35] Zhou W., Gao X., Liu D., Chen X. (2015). Chem. Rev..

[cit36] Samal A. K., Polavarapu L., Rodal-Cedeira S., Liz-Marzán L. M., Pérez-Juste J., Pastoriza-Santos I. (2013). Langmuir.

[cit37] He Y., Su S., Xu T., Zhong Y., Zapien J. A., Li J., Fan C., Lee S.-T. (2011). Nano Today.

[cit38] Li C.-Y., Meng M., Huang S.-C., Li L., Huang S.-R., Chen S., Meng L.-Y., Panneerselvam R., Zhang S.-J., Ren B., Yang Z.-L., Li J.-F., Tian Z.-Q. (2015). J. Am. Chem. Soc..

[cit39] Shao L., Zhang A., Rong Z., Wang C., Jia X., Zhang K., Xiao R., Wang S. (2018). Nanomedicine.

[cit40] Wang C., Li M., Li Q., Zhang K., Wang C., Xiao R., Wang S. (2017). RSC Adv..

[cit41] Wang J., Wu X., Wang C., Shao N., Dong P., Xiao R., Wang S. (2015). ACS Appl. Mater. Interfaces.

[cit42] Wada A., Sakoda Y., Oyamada T., Kida H. (2011). J. Virol. Methods.

[cit43] Wiriyachaiporn N., Maneeprakorn W., Apiwat C., Dharakul T. (2014). Microchim. Acta.

[cit44] Wu F., Yuan H., Zhou C., Mao M., Liu Q., Shen H., Cen Y., Qin Z., Ma L., Song Li L. (2016). Biosens. Bioelectron..

[cit45] Bamrungsap S., Apiwat C., Chantima W., Dharakul T., Wiriyachaiporn N. (2013). Microchim. Acta.

