# Supplementary material for: Dual dye-loaded Au@Ag coupled to a lateral flow immunoassay for the accurate and sensitive detection of Mycoplasma pneumoniae infection
Source: RSC Adv. 2018 Jun 11;8(38):21243–51. doi: 10.1039/c8ra03323d (PMC9080884; doi:10.1039/c8ra03323d)
Supplement: RA-008-C8RA03323D-s001 [file RA-008-C8RA03323D-s001.pdf]

Electronic Supplementary Material (ESI) for RSC Advances.
This journal is © The Royal Society of Chemistry 2018

Supplementary Information

Dual dye-loaded Au@Ag coupled to a lateral flow immunoassay for the accurate and sensitive detection of *Mycoplasma pneumonia* infection

Xiaofei Jia,‡[ab] Chongwen Wang,‡[ab] Zhen Rong,‡[b] Jian Li,[c] Keli Wang,[b] Zhiwei Qie,[b] Rui Xiao*[b] and Shengqi Wang*[ab]

[a] College of Life Sciences & Bio-Engineering, Beijing University of Technology, Beijing 100124, PR China.

[b] Beijing Institute of Radiation Medicine, Beijing 100850, PR China.

[c] Chinese PLA General Hospital, Beijing 100853, PR China.

* Corresponding authors. Tel: +86-10-66932211. E-mail: sqwang@bmi.ac.cn.

Tel: +86-10-66931422-5. E-mail: ruixiao203@sina.com.

† Electronic supplementary information (ESI) available.

‡ These authors contributed equally to this work.

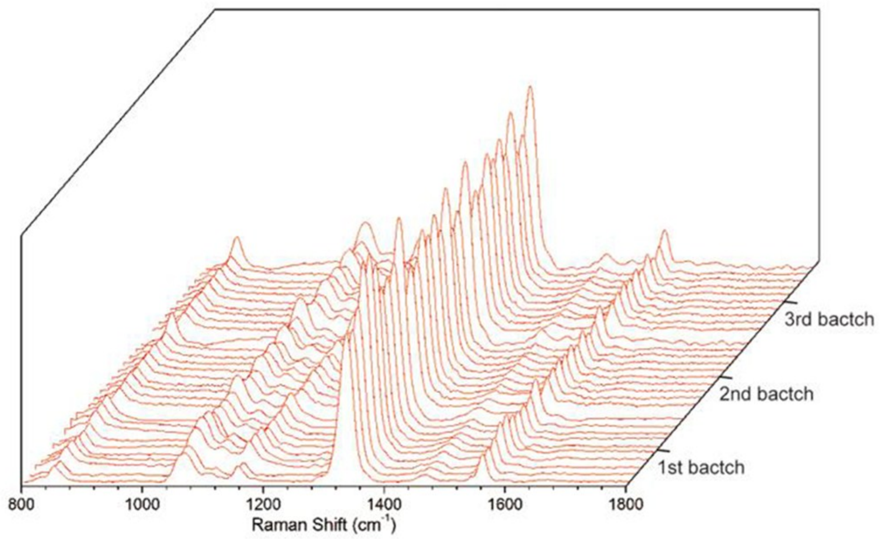

Figure S1. The repeatability of the proposed SERS-LFIA strips for human IgM detection (10 ng/mL). The SERS spectra collected randomly from 10 spots on the three different batches of SERS-LFIA strips.

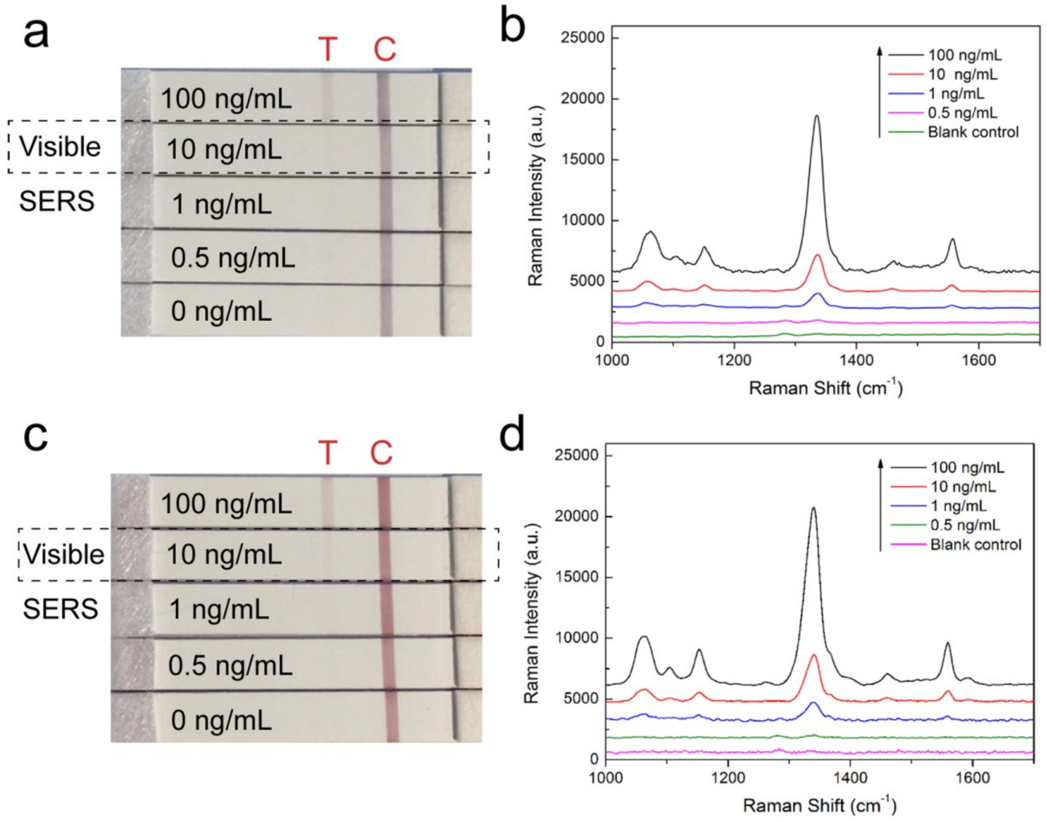

Figure S2. Photographs of the SERS-LFIA strips based on Au/DTNB (a) and Au@Ag/DTNB NPs (c) as the SERS tags after applying different concentrations of human IgM. (b) and (d) Corresponding SERS spectra obtained from the T line of (a) and (c), respectively. The integration times were 5 s and the excitation laser energy

was 10 mW.

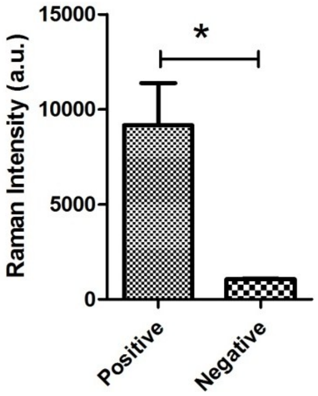

Figure S3. Comparison of positive group (20 MP-specific IgM positive serum specimens) and negative group (10 MP-specific IgM negative serum specimens) based on SERS-LFIA strips. *$p < 0.05$.

Table S1. Characteristics of the SERS-based LFIA strip developed in this work compared to other recently reported LFIA strip for respiratory pathogens detection.

Strategy	Respiratory pathogen	Limit of detection (LOD)	Tested sample	Reference
Fluorescence	Influenza A (H1N1)	250 ng·mL^{-1}	Allantoic fluid	Suwussa Bamrungsap et al
Au NPs	Influenza A (H1N1)	47 $TCID_{50}$ mL^{-1}	Influenza A infected MDCK cells	Natpapas Wiriyachaiporn et al
Au NPs with silver amplification	Influenza A (H5N1)	0.5 ng mL^{-1}	Allantoic fluid	Atsuhiko Wada et al
Quantum dot	Influenza A (H9N2)	0.25 HAU	Human serum	Feng Wu et al
SERS	Influenza A (H1N1)	6.7 ng mL^{-1}	Allantoic fluid	Maneeprakorn et al
SERS	Mycoplasma pneumoniae	0.1 ng mL^{-1}	Human serum	This work
